# The Circadian Variation in Anti-Müllerian Hormone in Patients with Polycystic Ovary Syndrome Differs Significantly from Normally Ovulating Women

**DOI:** 10.1371/journal.pone.0068223

**Published:** 2013-09-04

**Authors:** Leif Bungum, Florencia Franssohn, Mona Bungum, Peter Humaidan, Aleksander Giwercman

**Affiliations:** 1 Reproductive Medicine Center, Skåne University Hospital, Lund University, Malmö, Sweden; 2 Fertility Clinic, Odense University Hospital, Odense, Denmark; University of Kansas Medical Center, United States of America

## Abstract

**Obective:**

To improve the biologic understanding of the Polycystic Ovarian Syndrome (PCOS) condition by examining the circadian variation and relationship between Anti Müllerian Hormone (AMH), gonadotropins and ovarian steroids in PCOS patients compared to normally ovulating and menstruating women. By comparing the pattern of co-variation between AMH and Luteinizing Hormone, two compounds closely linked to hyperandrogenism and anovulation in PCOS, the involvement of the Hypothalamic-Pituitary-Ovarian axis in PCOS pathology could be elucidated.

**Patients:**

Eight normal-weighted young, anovulatory PCOS-women as study group and ten normal menstruating and ovulating women as controls.

**Interventions:**

Observational prospective study of the circadian variation in AMH, gonadotropins, sex steroids and androgens in a study and a control group. A circadian profile was performed in each study and control subject during a 24-h period by blood sampling every second hour, starting at 8:00 a.m. and continuing until 8:00 a.m. the following day.

**Result(s):**

Significant differences in hormonal levels were found between the groups, with higher concentrations of AMH, LH and androgens in the PCOS group and lower amounts of FSH and progesterone. A distinct difference in the circadian variation pattern of AMH and LH between PCOS patients and normal controls was seen, with PCOS patients presenting a uniform pattern in serum levels of AMH and LH throughout the study period, without significant nadir late-night values as was seen in the control group. In PCOS women, a significant positive association between LH/ FSH and testosterone was found opposite to controls.

**Main outcome measures:**

Circadian variation in Anti-Müllerian Hormone, gonadotropins and ovarian steroids and the covariation between them.

**Conclusion:**

A significant difference in the circadian secretion of LH and AMH in PCOS women compared to normally ovulating women indicate an increased GnRH pulse, creating high and constant LH serum concentrations. A significant co-variation between LH and AMH may suggest LH as a factor involved in the control of AMH secretion.

## Introduction

Polycystic ovary syndrome (PCOS), anovulation and clinical or biochemical hyperandrogenism, are phenotypically heterogenic endocrine disorders, affecting women of reproductive age with a prevalence of 6–10% [1]. Obesity, insulin resistance and the metabolic syndrome may also be related to PCOS.

Polycystic ovaries as a central feature of PCOS are secondary to follicular arrest interfering with normal folliculogenesis, including follicle recruitment, follicle dominance and ovulation. Although there is no consensus as to an explanation of the biological mechanisms behind PCOS, the condition seems to be at least two-factorial [2]. Firstly, the intra-ovarian hyperandrogenism promotes early follicular growth and leads to an excess in follicles measuring from 2–5 mm. Secondly, a low aromatase activity caused by insufficient Follicle Stimulating Hormone [3] activity impairs estrogen synthesis interfering with the selection and growth of a dominant follicle [4]. Insulin resistance, secondary to both genetic and lifestyle related factors as e.g. overweight is associated with anovulation, but is probably not the primary cause of PCOS [5]–[8]. Androgen production is driven by Luteinizing Hormone (LH)-stimulated steroidogenesis in theca interna cells [9] and hyperandrogenism may have both an extra- and intra-ovarian origin. An increased pituitary output of LH secondary to an altered Gonadotropin Releasing Hormone (GnRH) pulse [10] may be reinforced by other PCOS related factors like hyperinsulinemia, triggering a reduction of SHBG levels and enhanced bioavailability of free testosterone. Actually, insulin has been reported to increase LH secretion secondary to altered GnRH-neurone activity in both animals and in normally menstruating women [11], but this issue is debated due to surveys of insulin-infusion in PCOS-women not confirming this effect [12]–[15]. If present, both mechanisms would promote an increase in the androgen synthesis [16], [17] which may further deteriorate the regulation of the folliculogenesis.

Androgens induce polycystic ovaries in primates [18], and raised insulin levels secondary to insulin resistance increases the ability of the granulosa cells to respond to LH which may cause follicular arrest [19]. A positive correlation between androgen levels and the amount of follicles in an ovary has previously been reported [20], [21] and anti-androgen-therapy reduces the excess amount of follicles in PCOS patients [22]. Finally, an up-regulation of gene transcription and paracrine actions from granulosa cell derived Inhibins [23], [24] and Anti-Müllerian Hormone (AMH) [25], may deteriorate the de-regulated androgen biosynthesis [9], [26] through aromatase-inhibition, slowing down the conversion of androgens to estradiol.

Anti-Müllerian Hormone, a member of the transforming growth-factor β family (TGFβ) reduces the follicle sensivity to FSH and limits the primordial follicle transition into growing follicles [27]. Levels of AMH are usually two- to threefold higher in women diagnosed with PCOS compared to normally ovulating and menstruating women, and the increased AMH-concentration is positively correlated to the androgen level [25], [28]–[30].

Recently, we reported a significant circadian variation in AMH levels and a significant positive correlation between AMH and LH levels in normally menstruating women [31]. In order to improve our understanding of the biology of the PCOS condition and since AMH and LH seem closely linked to hyperandrogenism and anovulation in PCOS, it would be of interest to examine whether the pattern of co-variation between AMH and LH, seen in normally ovulating women, is preserved in those patients suffering from PCOS. Therefore, the aim of this study was to explore the circadian variation and relationship between AMH, gonadotropins and ovarian steroids in PCOS patients compared to normally ovulating and menstruating women.

## Materials and Methods

### Study design

This was an observational prospective study of the circadian variation in AMH, gonadotropins, sex steroids and androgens in PCOS patients compared to a control group, consisting of normally ovulation women. The study was conducted at the Reproductive Medicine Centre at Skane University Hospital, Malmo, Lund University, Sweden.

### Study group

Patients diagnosed with PCOS according to the Rotterdam criteria [32] and identified through the ICD-10 diagnosis code (E28.2) in RMC's electronic medical file system were invited to participate in the study. Exclusion criteria were pregnancy or on-going treatment with either gonadotropins, estrogens/progestins or the use of tobacco. None of the subjects had galactorrhea or any endocrine or systemic diseases, apart from PCOS, which might impact their reproductive physiology.

Patients were informed about the study either by written information or orally at the out-patient clinic.

Seventy-eight women were invited to take part in the study and 25 expressed interest of participating. Nine patients were excluded, five of them due to pregnancy, one due to estrogen treatment, and three due to smoking. Among the remaining 16 women who all underwent blood sampling throughout a 24- hour period, twelve turned out to be anovulatory defined as oligo-amenorrhea with fewer than eight menstrual bleedings per year, occurring at intervals longer than 35 days [33]. In order to achieve a match with the controls, eight of these 12 subjects aged below 30 years, having a BMI below 30 were defined as the study group. Their mean age was 24,6 years, median 25,0 was (range 16–29) and mean BMI was 23,2 kg/m^2^, median 22,5 (range 20–27). Table 1 shows the background characteristics of the participants.

**Table 1:** Demographic characteristics over study subject

	No	Age (mean)	Age (median) (range)	Body Mass Index (kg/m2) (mean) (SD)	Menstrual cycle characteristics Median (days)(range)
Study group (PCOS)	8	24,6 (3,8)	25 (16–29)	23,5 (2,9)	irregular/amenoroic
Control group	10	26 (1,7)	26,1 (22–29)	21,8 (2,5)	28,5 (22–35)

### Control group

Ten healthy women aged 20–30 years who previously participated in a study of the circadian variation of AMH in normally ovulating women [31] served as controls. The study subjects were enrolled by recruitment posters or advertisement in the local newspapers. They were non-smokers and had no history of infertility, hormonal medication or gynecological and chronic diseases; all presented with a Body Mass Index (BMI) below 30 kg/m^2^. Moreover, the control group consisted of normo-ovulatory, regularly menstruating women with a cycle length of 21 to 35 days, and in the study cycle they all had a significant mid-luteal progesterone rise, indicative of ovulation. Details regarding recruitment of the control group have previously been published [31].

Both controls and PCOS women signed an informed written consent and the study was approved by the ethical committee at Lund's University, DNR 2011/321.

### Blood sampling

Blood sampling was initiated on one of days 2–6 of the menstrual cycle in controls and at a random day in PCOS women. The circadian profile was performed during a 24-h period by blood sampling every second hour, starting at 8:00 a.m. and continuing until 8:00 a.m. the following day.

Through a heparinized catheter inserted into a forearm vein, each blood sample consisted of 10 mL blood drawn into vacuumed vials containing gel. Within two hours, the samples were centrifuged at 2000g for 10 min, and serum was isolated and stored at −20^°^C. Assays were performed within in a period of 2 months.

### Assays

Serum AMH was analyzed, using the Immunotech EIA AMH/MIS assay from Beckman–Coulter Inc., Marseille, France [34]. The lowest detectable level, distinguishable from zero with 95% confidence is 0.7 pmol/l. The total coefficient of variations (CVs) obtained were 25% at 5.7 pmol/l and 12% at 52 pmol/l. For FSH, LH, progesterone and estradiol, all samples from one participant were analyzed within the same assay run at a Beckman Access Immunoassay System on a UniCelTMDxI800 from Beckman–Coulter Inc., Brea, CA, USA. The lowest detectable level, distinguishable from zero with 95% confidence and total CVs are 0.2 IU/l and <9% for FSH and LH, 0.25 nmol/l and <0,14% for progesterone and 73 pmol/l and <13% for estradiol. Sex Hormon-binding Globuline (SHBG) was analyzed by immunometric sandwich assay, intraassay CV 5,3%, interassay CV 8%. Serum value of total testosterone and androstendione were assayed by a competitive immunoassay with luminmetric technique, interassay CV 7%, interassay 10%. Free testosterone concentration, was calculated as recommended by Vermeulen *et* *al* [35]. Cortisol was analyzed by a one-step competitive Electro-Chemi-Luminiscence-Immunoassay (ECLI) detection method, with a limit of 0,5 nol/L and intraassay CV 2.1%.

### Statistical analysis

We performed mixed model analyses for the repeated measurements of AMH, LH, FSH, estradiol and progesterone that were considered to be independent continuous variables (continuous) modeled with Group (A/B) and time (all time points: 8:00 a.m., 10:00 a.m., 12:00 p.m., 2:00p.m., 4:00 p.m., 6:00 p.m., 8:00 p.m., 10:00 p.m., 12:00 a.m., 2:00 a.m., 4:00 a.m., 6:00 a.m. and 8:00 a.m.) as categorical variables. For AMH, LH, FSH, estradiol and progesterone, the analysis was performed with and without the other hormones as continuous co-variates. Mixed model analysis allows the evaluation of differences in repeated measurements between patient groups. Compared with more simple statistical methods, mixed model analysis compute the overall mean difference between the groups and the overall time pattern of the variance, and thereby avoids multiple testing at individual time points. Another advantage of this statistical method is that clinically important differences between patient groups under investigation can be adjusted for. Repeated measurements at different time points imply that measurements for the same patient are more similar than those for different patients, i.e. the residuals of the mixed model for repeated measurements within a patient will be correlated. This correlation was assumed to follow an autoregressive structure with one time lag. A random coefficient was kept in the model only if its estimated variance was non-zero. Group-specific circadian variations were estimated as marginal means. The mixed model analysis also allows a comparison of each single time point with the first value (8:00 a.m. on the first day), and thus computes a significance level for each time point throughout the blood sampling period.

The maximum relative intra-individual variations in AMH levels (difference between the highest value and the lowest value during the 24 hour period, as percentage of the latter) found in PCOS and control-subjects were compared using the Mann–Whitney test.

Statistical analysis was performed using statistical software (SPSS 17.0 for Windows; SPSS Inc., Chicago, IL, USA). A p-value of ≤0.05 was considered statistically significant.

## Results

### Circadian variation in AMH

**Figure 1:**
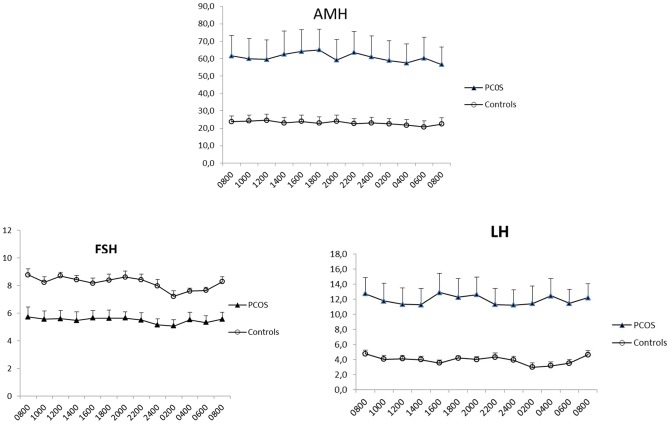
Circadian variation in AMH (pmol/L), LH (IU/L) and FSH (IU/L). Figures illustrate the mean values + SEM for PCOS and controls

A significant difference in mean AMH levels between the groups was observed, the highest values being seen in the PCOS group (P = 0,004). The mean difference was 37,1 pmol/L (95% CI: 31,0; 43,2 pmol/L). With 8:00 a.m. values on the first day of investigation as a reference, the mean concentrations in the study group revealed a statistically significant variation throughout the sampling period (p = 0.015). Unlike the control group, where significantly lower AMH values were seen in the early morning, the study group revealed no such uniform pattern with subjects having nadir values at different time points of the diurnal period (Table 2, Fig. 1).

**Table 2:** Serum concentration of AMH, FSH and LH in relation to the time of day and group.

Time	8.00am	10.00am	12.00pm	2.00pm	4.00pm	6.00pm	8.00pm	10.00pm	12.00am	2.00am	4.00am	6.00am	8.00am
**AMH**													
PCOS, pmol/L; mean (SD)	61,6 (35,1)	60,0 (35,1)	59,6 (33,9)	62,5 (40,2)	64,1 (38,0)	65,0 (35,6)	59,1 (36,2)	63,5 (36,8)	61,0 (36,0)	58,9 (34,0)	57,5 (32,9)	60,4 (36,2)	56,7 (30,8)
Controls, pmol/L, mean (SD)	23,8 (10,0)	24,2 (11,3)	24,5 (11,8)	23,0 (10,9)	23,9 (12,6)	22,9 (11,8)	24,0 (11,7)	22,6 (10,0)	23,0 (10,7)	22,6 (10,2)	21,7 (10,9)	20,8 * (11,3)	22,4 (12,0)
**FSH**													
PCOS, IU/L, mean (SD)	5,7 (1,2)	5,6 (1,1)	5,6 (0,8)	5,5 (0,9)	5,6 (1,1)	5,6 (1,2)	5,6 (1,3)	5,5 (1,2)	5,1 (1,3)	5,1* (1,2)	5,5 (0,6)	5,3 (0,7)	5,6 (1,0)
Controls, IU/L, mean (SD)	8,8 (3,1)	8,2 (2,6)	8,7 (2,6)	8,4 (2,7)	8,5 (2,3)	8,4 (2,5)	8,6 (2,0)	8,4 (2,3)	8,0 (1,9)	7,2* (1,9)	7,6* (2,4)	7,7* (2,4)	8,3 (2,2)
**LH**													
PCOS, IU/L, mean (SD)	12,8 (6,3)	11,8 (7,2)	11,3 (6,6)	11,3 (6,5)	12,9 (7,6)	12,3 (7,5)	2,6 (7,1)	11,3 (6,5)	11,2 (5,0)	11,4 (7,0)	12,5 (6,8)	11,5 (5,6)	12,2 (5,6)
Controls, IU/L, mean (SD)	4,8 (1,6)	4,1 (1,6)	4,1 (1,5)	4,0 (1,4)	3,8 (1,2)	4,2 (0,9)	4,0 (1,2)	4,3 (1,8)	3,9 (1,7)	3,0* (1,9)	3,2* (1,7)	3,5* (1,7)	4,7 (1,7)

* p<0,05 in comparison to 08.00 a.m. levels.

The relative median (range) maximum intra-individual variation in AMH concentration through the 24 hours period was 29% (13–63%) in the study group and 23% (10–230%) in the control group; this difference was not statistically significant.

### Circadian variation in gonadotropins

A significant difference in mean FSH levels between the groups was observed, the lowest values being found in the PCOS group (p = 0.005) (Table 2, Fig. 1). The mean difference was 2,7 IU/L (95% CI: −3,2; –2,2 IU/L).

The circadian variation in FSH in the PCOS group showed no significant variation over the 24-h period (p = 0,315), similar to findings of the control group (p = 0,075). The control group had a period of statistically significantly suppressed levels at 2 a.m., 4 a.m. and 6 a.m. while the study group showed significant nadir values at 2 a.m.

A significant difference in mean LH levels between the groups was observed, the highest values being found in the PCOS group (p = 0,001) with a mean difference between groups of 8,0 IU/L (95% CI: 6,7; 9,0 IU/L).

LH showed no variation by time in the PCOS group (p = 0,8) and no nadir nocturnal values unlike the control group which varied significantly throughout the 24 hour period (p = 0.045) displaying significantly lower values at 2 a.m., 4 a.m. and 6 a.m.

#### Androgens

**Figure 2:**
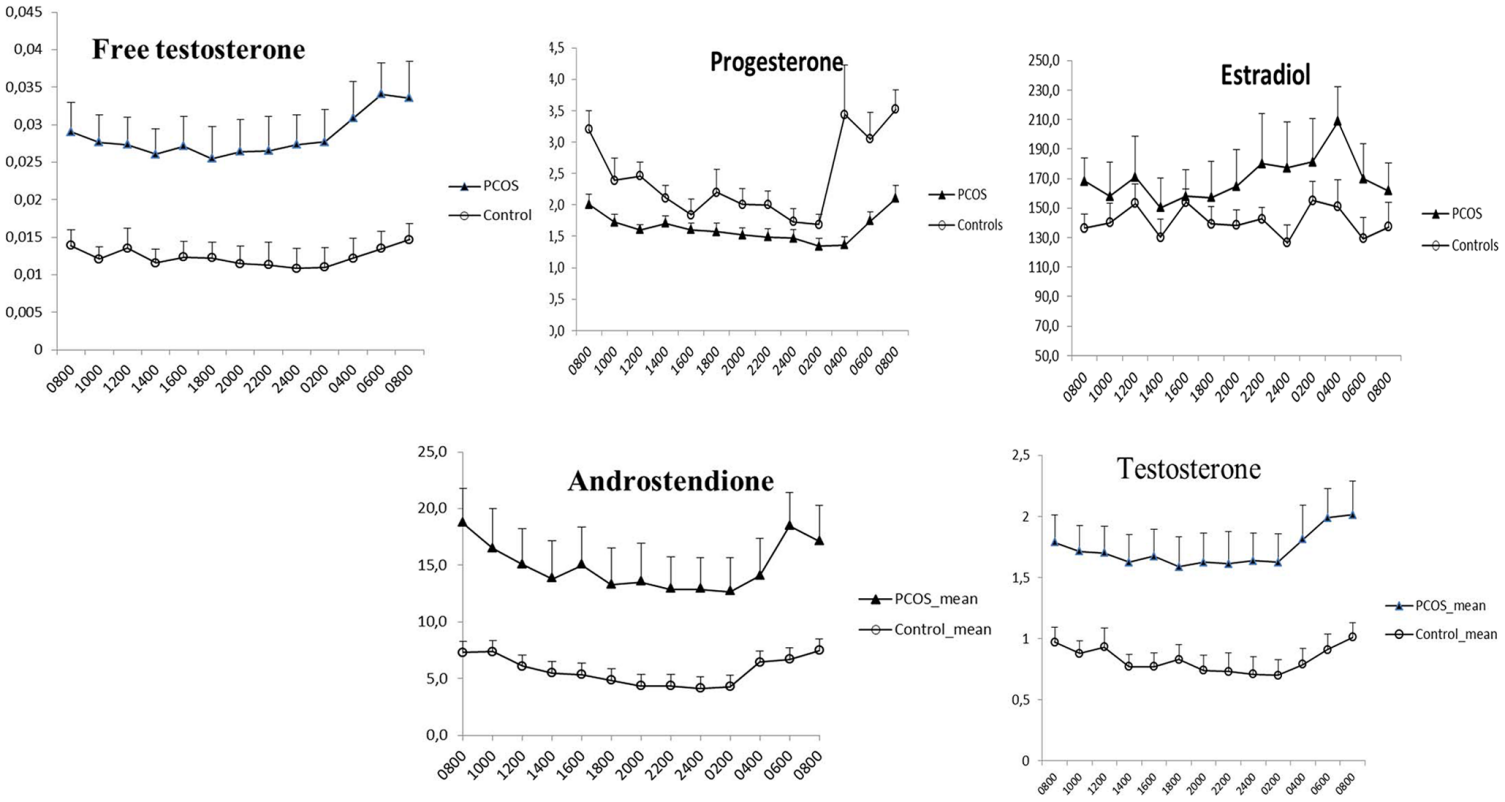
Circadian variation in Progesterone (nmol/L), Estradiol (IU/L), Testosterone, Androstendione and free Testosterone. Figures illustrate the mean values + SEM for PCOS and controls.

A significant difference between groups in levels of both androstendione: mean difference 9,3 nmol/L (95% CI: 2,98–15,52 nmol/L) p = 0.006 and testosterone: mean difference 0.89 nmol/L (95% CI: 0,34–1,46 nmol/L) p = 0.004 was observed, the highest values being seen in the PCOS group. Both androgens revealed a significant variation over time in both groups; androstendione (controls p = 0,005; PCOS p<0,001) and testosterone (controls p<0.001; PCOS p = 0.001) (Table 3, Fig. 2). Moreover, the free testosterone level was significantly higher in PCOS- women, p = 0.002, mean difference 0.016 nmol/L (5% CI: 0.007–0.026 nmol/L). The circadian variation was statistically significant in both groups; PCOS: p<0,0001; controls p = 0.02.

**Table 3:** Serum concentration of Testosterone, Androstendione and free Testosterone in relation to the time of the day and group.

Time	8.00am	10.00am	12.00pm	2.00pm	4.00pm	6.00pm	8.00pm	10.00pm	12.00am	2.00am	4.00am	6.00am	8.00am
**Testosterone**													
PCOS, pmol/ Lmean (SD)	1,8 (0,7)	1,7 (0,6)	1,7 (0,7)	1,6 (0,7)	1,7 (0,7)	1,6 (0,7)	1,6* (0,7)	1,6 (0,8)	1,6 (0,7)	1,6 (0,7)	1,8 (0,8)	2,0* (0,7)	2,0* (0,8)
Controls, pmol/ Lmean (SD)	1,0 (0,4)	0,9 (0,3)	0,9 (0,5)	0,8* (0,3)	0,8* (0,4)	0,8* (0,4)	0,7* (0,4)	0,7* (0,5)	0,7* (0,5)	0,7* (0,4)	0,8* (0,4)	0,9 (0,4)	1,0 (0,4)
**Androstendion**e													
PCOS, IU/ L, mean (SD)	18,7 (9,3)	16,5 (10,8)	15,1* (9,6)	13,8* (10,2)	15,0* (10,1)	13,3* (9,9)	13,5* (10,3)	12,9* (8,6)	12,9* (8,3)	12,7* (9,0)	14,1 (10,1)	18,5 (9,0)	17,1 (9,7)
Controls, IU/ L, mean (SD)	7,3 (2,0)	7,4 (4,4)	6,1 (1,8)	5,5* (1,0)	5,4* (1,2)	4,9* (1,1)	4,4* (1,2)	4,4* (1,4)	4,2* (0,9)	4,3* (1,7)	6,4 (2,8)	6,7 (3,6)	7,7 (2,4)
**Free Testosterone**													
PCOS, IU/ L, mean (SD)	0,029 (0,011)	0,028 (0,010)	0,027 (0,010)	0,026* (0,010)	0,027 (0,011)	0,025* (0,012)	0,026 (0,012)	0,027 (0,013)	0,027 (0,011)	0,028 (0,012)	0,031 (0,014)	0,034* (0,012)	0,034* (0,014)
Controls, IU/ L, mean (SD)	0,014 (0,006)	0,012 (0,005)	0,014 (0,008)	0,012* (0,005)	0,012* (0,006)	0,012 (0,006)	0,011* (0,007)	0,011* (0,008)	0,011* (0,007)	0,011* (0,007)	0,012 (0,007)	0,014 (0,006)	0,015 (0,006)

* p<0,05 in comparison to 08.00 a.m. levels.

For testosterone levels, the morning value measured after 24 hours was marginally different compared to baseline.

### Circadian variation in ovarian-derived hormones

Progesterone levels were significantly lower in the PCOS-group (p = 0.,007) compared to the control group. The mean difference was −0,78 nmol/L (95% CI: −1.0; –0.51 nmol/L). The mean progesterone levels revealed a rapid fall during the daytime, when compared to the initial 8 a.m. measurement and increased again in the early morning. These variations were highly significant in the both the PCOS and the control group (p<0.001) (Table 4, Fig. 2). In contrast, the estradiol levels did not vary over time in neither study subjects nor controls and there was no difference between the groups (p = 0,3).

**Table 4:** Serum concentration of Progesterone and Estradiol in relation to the time of the day and group.

Time	8,00m	10,00am	12,00pm	2,00pm	4,00pm	6,00pm	8,00pm	10,00pm	12,00am	2,00am	4,00am	6,00am	8,00am
**Progesterone**													
PCOS, nmol/L, Mean (SD)	2,0 (0,5)	1,7* (0,4)	1,6* (0,3)	1,7* (0,4)	1,6* (0,4)	1,6* (0,4)	1,5* (0,3)	1,5* (0,4)	1,4* (0,4)	1,3* (0,4)	1,4* (0,4)	1,7* (0,4)	2,1 (0,6)
Controls, nmol/L, mean (SD)	3,2 (1,0)	2,3 (1,2)	2,4 (0,8)	2,2* (0,7)	2,0* (0,8)	2,3 (1,2)	2,0* (0,8)	1,9* (0,8)	1,6* (0,7)	1,6* (0,6)	3,3 (2,6)	3,1 (1,4)	3,6 (1,0)
**Estradiol**													
PCOS, pmol/L, mean (SD)	168,1 (47,7)	158,1 (70,0)	170,9 (84,3)	150,4 (60,0)	157,9 (54,5)	157,2 (73,6)	164,7 (75,0)	180,1 (102,4)	140,8 (58,7)	181,4 (88,4)	208,7 (71,5)	169,9 (72,0)	161,6 (57,2)
Controls, pmol/L, mean (SD)	136,4 (29,8)	140,0 (41,1)	153,1 (40,9)	130,1 (39,6)	157,9 (27,6)	139,1 (37,1)	138,4 (32,6)	142,6 (24,9)	126,3 (39,2)	155,2 (41,5)	150,8 (57,5)	129,4 (44,5)	137,2 (53,0)

* p<0,05 in comparison to 08.00 a.m. levels.

### Circadian variation in SHBG

In PCOS women, a statistically significant circadian variation in SHBG was found (p<0,0001). Significantly lower values compared to the first measurements were reached by midnight (p = 003), and values continued to fall with an absolute nadir at 0600 a.m (p<0,0001). This variation was not seen in controls (p = 0,089). Moreover, no significant differences in SHBG levels were seen between the two groups.

### Circadian variation in cortisol

No difference was found in cortisol levels between controls and PCOS women (p = 0,3). Both groups had a highly significant variation over time with nadir values at 0200 a.m. (p<0, 0001) in both groups.

### Co-variation between serum levels of AMH and other reproductive hormones

A statistically significant positive co-variation was found between AMH and LH, which applied to both PCOS women p = 0.001, 0,12 (95% CI: 0,06; 0;19) and the control group p = 0,002, 0,06 (95% CI: 0,03; 0,10). No such association was found between the variation in AMH and any other of the hormones measured, including progesterone, estradiol, androgens and cortisol.

### Co-variation between serum levels of gonadotropins and progesterone/estradiol

A statistically significant positive co-variation was found between LH and progesterone, seen in both groups, PCOS p = 0,001, 0,02 (95% CI: 0,01; 0,03) and controls p = 0,001, 0,26 (95% CI: 0,11–0,40). The same was true for the co-variation between FSH and progesterone (both groups p<0,001), PCOS 0,15% (CI: 0,08; 0,21) and controls 0,20 (95% CI: 0,10; 0,19). There was, however, no co-variation between LH and estradiol.

### Co-variation between serum levels of gonadotropins and androgens

Androstendione did not correlate to FSH and LH. In the control group, no significant co-variation between LH/ FSH and androstendione/testosterone/free testosterone was found. However, PCOS-women demonstrated a highly significant co-variation between LH and testosterone p = 0,0001, 0,02 (95% CI: 0,01; 0,04) and a modest association with free testosterone p = 0,023, 0,0002 (95% CI: 0,0003; 0,0005). Between FSH and testosterone levels a positive associations was also found p = 0,03, 0,09 (95% CI: 0,01; 0,17); this was not the case for free testosterone (p = 0,3).

## Discussion

This is the first study to explore the circadian variation in AMH in PCOS women and its co-variation with gonadotropins and ovarian steroids. The major finding of our study was a difference in the circadian variation pattern of AMH and LH between PCOS patients and normal controls. Unlike controls, a uniform pattern of variation in serum levels of AMH and LH without significant nadir late-night values was seen in the PCOS group. A significant positive co-variation between AMH and LH was seen in both groups. However, in PCOS women, a significant positive association between LH/ FSH and testosterone was found which was not the case in controls.

Pulsatile GnRH-secretion plays an essential role in the neuroendocrine control of reproductive function, and a distorted gonadotropin-secretion is a hallmark of PCOS [33], [36]–[38]. The higher LH-level and lower FSH is believed to be secondary to an abnormal neuroendocrine function with an altered GnRH-pulse probably under the influence of positive feed-back effects from ovarian steroids and androgens, resulting in excess LH and low FSH [33]. Moreover, an excess level of LH receptors has been found both on theca and granulosa-cells from PCOS women [39] who were also reported to overexpress a different receptor genotype [40]. Additionally, studies in mammals have revealed increased LH activity, stimulating androgen secretion [41]; all features which possibly contribute to the pathogenesis of PCOS.

The study subjects included in this study revealed differences in their endocrine profile. Thus, the significantly higher level of AMH, LH, androstendione and testosterone (total and free fraction) in the study group compared to the controls, as well as a reduced FSH and progesterone level, are all findings characteristic of a PCOS cohort. Our study revealed no significant variation in LH in the circadian profile of PCOS women. In contrast, the control group had a significant late night hour reduction in LH levels, in accordance with earlier reports describing low follicular phase GnRH pulses in ovulatory women while PCOS subjects had constant and rapid pulses [42]. Such persistently rapid GnRH pulses favor the synthesis and secretion of LH over FSH, and probably depicts an insufficiency in the negative feedback systems necessary to suppress the GnRH-pulse generator rather than representing an acceleration of the pulse generator [37]. Our findings of a non-significant variation in LH are in accordance with more rapid GnRH-pulses producing a high LH level without significant variation and nadir values throughout the night. Low progesterone, as found in our study group, supports the assumption of a rapidly working GnRH pulse generator secondary to hyperandrogenemia affecting hypothalamic sensitivity to the pace-reducing effects of ovarian steroids [22], [37]. The significant co-variation between LH and progesterone underlines the reciprocal relationship between these two hormones.

We found a significantly lower FSH level in PCOS compared to controls, but no co-variation between AMH and FSH was noticed in the groups. Available reports on the relationship between AMH and FSH are inconsistent. In the human testis a well-established positive relationship exists between AMH gene expression and FSH [43], and in rat ovaries FSH has been reported to down-regulate AMH and AMH type II receptors [44]. Low FSH and estradiol in combination with high AMH levels have been reported by others. However, these studies [25], [45] did not reveal lower estradiol levels in combination with low FSH. Moreover, women undergoing IVF-treatment were reported to display a negative association with FSH, suggesting that the AMH-level was predictive of the FSH-level. However, this finding was not confirmed in a study including 200 PCOS-patients and 50 normo-ovulatory controls [46]. Thus, a reliable hypothesis on the possible biological mechanisms of such a relationship is still lacking. The significant positive co-variation between AMH and LH is interesting and this has been described by others. Moreover, a link between these two hormones has been shown both *in vitro* and *in vivo.* It has been postulated that the relationship between AMH and gonadotropins depends on the size of the ovarian reserve [47], based on a strong correlation between LH and AMH in young women with normal FSH and excess ovarian reserve while in subjects with high FSH marking reduced ovarian reserve, AMH and FSH was correlated [48]–[51]. This is well in accordance with our findings in a previously published study based on a normal ovulatory population of different ages [31], in whom a significant co-variation between AMH and LH but not AMH and FSH was found.

The present study revealed no correlation between AMH and androgens in either group. However, in PCOS women, a highly significant co-variation was found between LH and testosterone, but not with androstendione. As the latter hormone has both ovarian and adrenal origin, this might point towards the ovary as the central organ in this connection, since testosterone is entirely synthesized here. Furthermore, as AMH co-variates with LH, but not testosterone and LH co-variates strongly with testosterone, LH seems to be the controlling factor, especially in PCOS women.

In experimental animal studies, the AMH/LH relation has been linked up to the hypothalamic-pituitary-gonadal axis, implying AMH actions at the level of the pituitary [52]. In more recent studies exploring rat pituitary gonadotrope-deriven cell lines in culture, AMHR2 receptors and an AMH-induced enhanced transcription of FSH and LH β sub-units [53] were found. Although an interesting observation, findings in cultured cell lines may characterize the property of cells, but can hardly permit wide assumptions concerning their function in complex physiological scenery.

In our previous study [31] we were unable to conclude whether the variation in AMH levels drives the fluctuations in LH or vice versa, and as a third option we suggested a joint factor regulating the secretion of both hormones. In the present study, PCOS women had high, non-fluctuating diurnal levels of LH and AMH, indicating an increased activity in the GnRH pulse generator. Adding the finding of a strong correlation between LH and Testosterone, but not between AMH and Testosterone, could suggest a cascade of characteristics in PCOS women starting with an abnormal GnRH pulse and LH as a potential regulator of the AMH secretion.

On day 2 the morning testosterone value was slightly higher than the baseline concentration at the start of 24 hour period. This might be due to analytical variations, but could also be related to circumstances around the blood sampling - the awake period being longer before the first morning sampling as compared to the blood sample on the second day. Thus, an impact of the duration of the awake period on baseline parameters has previously been reported [54].

The weakness of the present study is the relatively limited number of study subjects fulfilling the inclusion criteria (age, hormonal status, clinical symptoms and BMI). On the other hand, the statistically significant associations found seem to be of a sufficient magnitude to draw conclusions even though the sample size is small. Moreover, the strength of the study is the frequent sampling throughout a 24 hour period in a PCOS study group.

In conclusion, we found a significant difference in the circadian secretion of LH and AMH in PCOS women compared to normally ovulating women. This may be explained by an increased GnRH pulse, creating high and constant LH serum concentrations. Moreover, a significant co-variation between LH and AMH was seen, suggesting LH as a possible factor involved in the control of AMH secretion. Future studies in PCOS women with different phenotypes are needed to validate our findings.
